# Deciphering the Molecular Characteristics of Human Idiopathic Nonobstructive Azoospermia from the Perspective of Germ Cells

**DOI:** 10.1002/advs.202206852

**Published:** 2023-04-21

**Authors:** Yidong Chen, Xixi Liu, Li Zhang, Feiyin Zhu, Liying Yan, Wenhao Tang, Zhe Zhang, Qiang Liu, Hui Jiang, Jie Qiao

**Affiliations:** ^1^ Center for Reproductive Medicine Department of Obstetrics and Gynecology Peking University Third Hospital Beijing 100191 China; ^2^ National Clinical Research Center for Obstetrics and Gynecology Beijing 100191 China; ^3^ Key Laboratory of Assisted Reproduction (Peking University) Ministry of Education Beijing 100191 China; ^4^ Peking‐Tsinghua Center for Life Sciences Peking University Beijing 100871 China; ^5^ Department of Urology Peking University Third Hospital Beijing 100191 China; ^6^ Beijing Key Laboratory of Reproductive Endocrinology and Assisted Reproductive Technology Beijing 100191 China; ^7^ Beijing Advanced Innovation Center for Genomics Beijing 100871 China

**Keywords:** clinical diagnosis, germ cells, molecular characteristics, nonobstructive azoospermia, pathogenesis, spermatogenic capacity predictor

## Abstract

Nonobstructive azoospermia (NOA) is one of the most important causes of male infertility, accounting for 10–15% of infertile men worldwide. Among these, more than 70% of cases are idiopathic NOA (iNOA), whose pathogenesis and molecular basis remain unknown. This work profiles 3696 human testicular single‐cell transcriptomes from 17 iNOA patients, which are classified into four classes with different arrest periods and variable cell proportions based on the gene expression patterns and pathological features. Genes related to the cell cycle, energy production, and gamete generation show obvious abnormalities in iNOA germ cells. This work identifies several candidate causal genes for iNOA, including *CD164*, *LELP1*, and *TEX38*, which are significantly downregulated in iNOA germ cells. Notably, *CD164* knockdown promotes apoptosis in spermatogonia. Cellular communications between spermatogonial stem cells and Sertoli cells are disturbed in iNOA patients. Moreover, *BOD1L2*, *C1orf194*, and *KRTCAP2* are found to indicate testicular spermatogenic capacity in a variety of testicular diseases, such as Y‐chromosome microdeletions and Klinefelter syndrome. In general, this study analyzes the pathogenesis of iNOA from the perspective of germ cell development, transcription factor (TF) regulatory networks, as well as germ cell and somatic cell interactions, which provides new ideas for clinical diagnosis.

## Graphical abstract

Based on the gene expression patterns and pathological features, idiopathic nonobstructive azoospermia (NOA) patients are classified into four classes. Transcriptional patterns are altered in idiopathic NOA patients, especially in cell cycle activity, energy production, and cellular communications between germ cells and somatic cells. A set of predictive genes is identified to indicate testicular spermatogenic capacity, which may be helpful for clinical diagnosis.

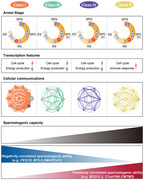

## 1 Introduction

Spermatogenesis is a precisely regulated and highly ordered process, including mitotic proliferation of spermatogonia, meiosis of spermatocytes, and differentiation of haploid spermatids during spermiogenesis.^[^ ^1^ ^]^ Any errors in this process will lead to male reproductive disorders. Male infertility, accounting for 50% of infertile couples, is a serious reproductive health problem.^[^ ^2^ ^]^ Nonobstructive azoospermia (NOA) is the most serious form, accounting for 10–15% of male infertility.^[^ ^3^ ^]^

Only one in three cases of azoospermia can be explained by genetic defects, such as abnormalities and deletion of the azoospermia‐factor (AZF) region in the Y chromosome, whereas more than 70% of the cases are idiopathic NOA (iNOA).^[^ ^4^, ^5^, ^6^, ^7^ ^]^ In addition to genetic defects, infectious and inflammatory conditions in the reproductive system contribute to male infertility.^[^ ^8^, ^9^, ^10^ ^]^ Genome‐wide association studies of idiopathic male infertility have revealed a series of NOA susceptibility loci, including *PEX10*, *PRMT6*, and *SIRPA*.^[^ ^11^, ^12^ ^]^ Several single‐gene mutations have been reported in patients with testicular‐phenotype NOA, including *TEX11*, *TEX14*, *TEX15*, *DMC1*, and *MEIOB*.^[^ ^13^, ^14^, ^15^, ^16^, ^17^ ^]^ Recent studies have used transcriptome analysis to investigate the pathogenic mechanism of NOA. Bulk transcriptome analyses have revealed that *PILRA* and *ZNF676* are related to NOA susceptibility.^[^ ^18^ ^]^ Two‐dimensional coculture of multiple stem cell types and three‐dimensional testicular organ culture have been used to simulate the environment of testicular sperm production and promote spermatogenesis, with promising results for the treatment of azoospermia.^[^ ^19^, ^20^, ^21^ ^]^

Treatments for azoospermia require a better understanding of the classification of azoospermia. Single‐cell RNA sequencing (scRNA‐seq) enables the study of highly heterogeneous cell populations in the testes at a single‐cell resolution. We previously conducted scRNA‐seq analysis of adult human testis samples from normal donors and iNOA patients and reconstructed the transcriptional programs inherent to sequential cell fate transition during human spermatogenesis. The results showed that the gene expression patterns of somatic cells in iNOA differed from those in normal spermatogenesis and mainly included DNA damage genes and other stress‐responsive genes.^[^ ^22^, ^23^ ^]^ A recent study revealed that maturation disorders in Sertoli cells are involved in iNOA, and inhibition of the Wnt signaling pathway promoted the maturation of these cells.^[^ ^24^ ^]^ However, the number of reported study cases is very small, and the explanation of the iNOA mechanism is limited from a somatic perspective.^[^ ^25^, ^26^ ^]^ The gene expression characteristics of germ cells and somatic cells, cell–cell interactions between germ cells and somatic cells, as well as the regulatory networks of transcription factors (TFs) in iNOA patients remain largely unknown.

In this study, we performed scRNA‐seq analysis of 3696 single cells from 17 iNOA patients with the aim of uncovering the pathogenic genes, networks, and pathogenesis of iNOA. We found that the 17 iNOA patients were classified into four classes (Classes I‐IV) with different arrest periods and variable cell proportions based on the gene expression patterns and pathological features. Genes related to the cell cycle were upregulated, and genes involved in energy metabolism and gametogenesis were downregulated in iNOA germ cells. *CD164*, *LELP1*, and *TEX38* with decreased expression in germ cells may be the causal genes of iNOA. Interestingly, *CD164* knockdown caused abnormal apoptosis of spermatogonia. Cell–cell interactions between spermatogonial stem cells and Sertoli cells dynamically changed in the iNOA state. Furthermore, a series of tendency genes were found to indicate testicular spermatogenic capacity in several testicular diseases. Overall, our work focused on revealing the pathogenesis of iNOA from the perspective of germ cells, and providing help for the clinical diagnosis and treatment of azoospermia.

## 2 Results

### 2.1 Gene Expression Patterns in Testicular Cells of iNOA Patients

**Figure 1:**
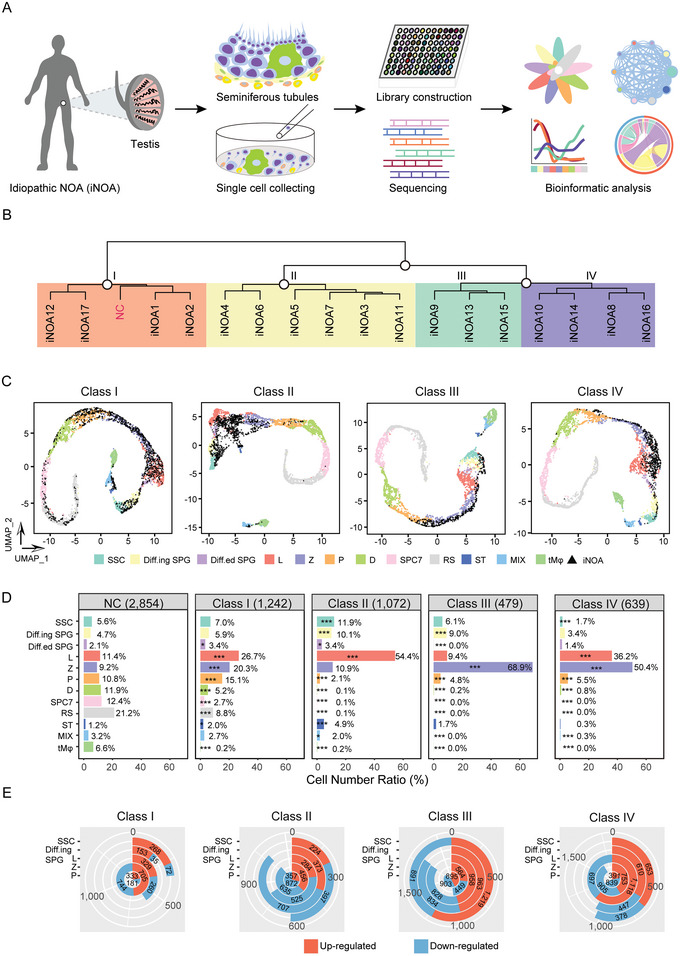
Global expression profiling of testicular cells from idiopathic nonobstructive azoospermia (iNOA) patients by scRNA‐seq. A) Schematic illustration of the workflow. B) Classification of the 17 iNOA patients based on the gene expression patterns and pathological features. C) UMAP plot of the four classes iNOA and normal controls (NC). Different colors indicate different cell types of NC, and testicular cells from iNOA patients are indicated in black. SSC, spermatogonial stem cells; Diff.ing SPG, differentiating spermatogonia; Diff.ed SPG, differentiated spermatogonia; L, leptotene spermatocytes; Z, zygotene spermatocytes; P, pachytene spermatocytes; D, diplotene spermatocytes; SPC7, spermatocyte 7; RS, round sperm; ST, Sertoli cells; MIX, mixture of peritubular myoid cells and Leydig cells; tM*φ*, testicular macrophages. D) Cell number ratio of testicular cells from NC and the four classes iNOA. The chi‐square test was used for the significance test. E) Differentially expressed genes (DEGs) between the four classes iNOA. Upregulated and downregulated genes are indicated in red and blue, respectively.

To characterize the cell‐type composition and molecular features of iNOA, we obtained 3696 testicular single cells in total from 17 iNOA patients and performed single‐cell RNA sequencing (**Figure** **1**A). After filtering the cells with more than 2000 genes and more than 10 000 transcripts, we retained 3432 cells of good quality. On average, 6928 expressed genes and 103918 mRNA molecules were detected in each cell (Figure S1A; Table S1, Supporting Information). Based on the gene expression patterns and pathological features, we classified the 17 patients into four classes. iNOA1, 2, 12, and 17 were clustered with normal samples, and were defined as Class I. Class II comprised iNOA3‐7 and iNOA11; Class III comprised iNOA9, 13, and 15, whereas Class IV included iNOA8, 10, 14, and 16 (Figure 1B and Figure S1B, Supporting Information).

We individually compared the four classes of iNOA samples with normal samples to identify the arrest stage and used classical marker genes to identify the various cell types (Figure S1C,D, Supporting Information). Interestingly, spermatogenesis in Class I was very similar to normal spermatogenesis, as we observed a few round spermatids in Class I. In the other three classes, cells reached the pachytene stage, but not the later stages (Figure 1C). Next, we determined the proportion of cells in each stage for each cell type. Class I iNOA patients had all cell types, but the cell ratios varied from those in the normal controls (NC), with a low level of damage to spermatogenesis. Class II iNOA patients had more leptotene spermatocytes (L) than the NC. Class III iNOA patients had more zygotene spermatocytes (Z) than the NC. Class IV iNOA patients had fewer spermatogonia and higher proportions of L and Z (Figure 1D). To find the most important differences among the four classes of iNOA, which may reflect the stage when the difference occurred, we identified differentially expressed genes (DEGs) in the same cell type of each class (Table S2, Supporting Information). The largest difference between Class I and the other three classes was found in zygotene spermatocytes. Pachytene spermatocytes (P), spermatogonial stem cells (SSC), and L were largely distinct in Classes II, III, and IV, respectively (Figure 1E).

### 2.2 Transcriptional Characteristics of Germ Cells in iNOA Patients

**Figure 2:**
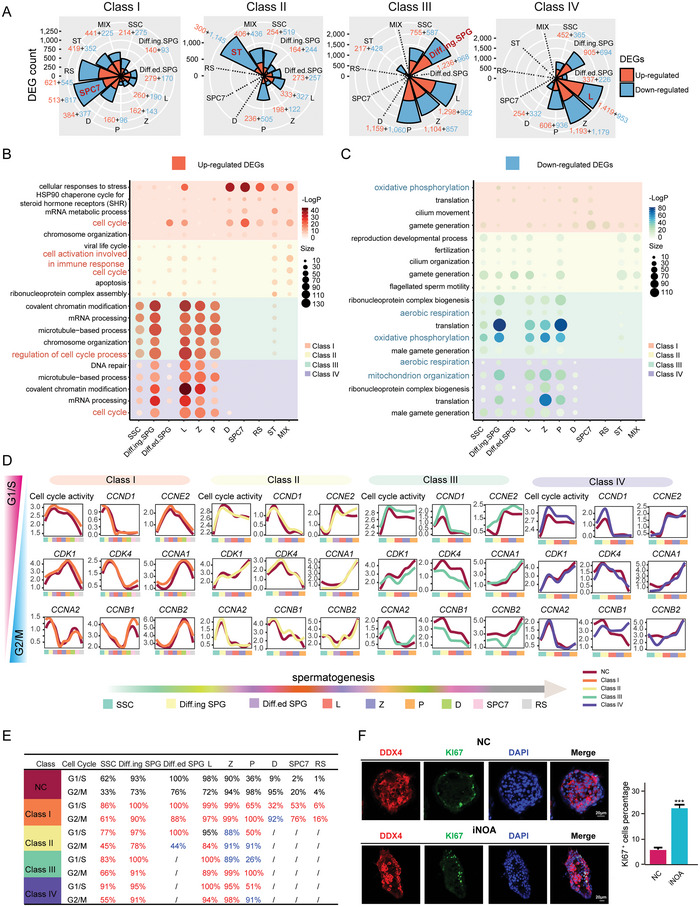
Transcriptional features of the four classes idiopathic nonobstructive azoospermia (iNOA). A) differentially expressed genes (DEGs) between normal controls (NC) and the four classes iNOA. B) Functions of the upregulated DEGs in the four classes iNOA. C) Functions of the downregulated DEGs in the four classes iNOA. D) Expression levels of G1/S and G2/M phase genes in the four classes iNOA. E) The ratio of cells with active cell cycle to total cells in each cluster according to the expression of G1/S and G2/M phase genes. F) Left panel, immunofluorescence of DDX4 (red, a marker for germ cell) costained with KI67 (green) in NC and iNOA. The scale bar represents 20 µm. Right panel, bar plot of the KI67 ^+^ cell percentage. A two‐tailed Student's t test was used for the significance test.

To find clues on the cause of iNOA, we identified DEGs between the four classes of iNOA and NC (Table S2, Supporting Information). Interestingly, spermatocyte 7 (SPC7), Sertoli cells (ST), differentiating spermatogonia (Diff.ing SPG), and L were the cell types that differed the most from normal spermatogenesis in four classes of iNOA, based on the number of DEGs (**Figure** **2**A). The functions of these DEGs varied among the four classes of iNOA. The genes upregulated in Class I iNOA were highly enriched in cellular responses to stress, mRNA metabolic process, and the cell cycle according to Gene Ontology (GO) analysis. In Class II, the upregulated genes were mostly associated with cell activation involved in immune response, cell cycle and apoptosis. Covalent chromatin modification, chromosome organization, and cell cycle were the most enriched GO terms in Class III iNOA. Similar to Class III, Class IV was highly enriched in chromosome organization, cell cycle, and DNA repair (Figure 2B). Some studies have shown that the occurrence of testicular germ cell apoptosis can affect the normal spermatogenesis.^[^ ^27^, ^28^ ^]^ Therefore, we examined the expression of apoptosis‐related genes in germ cells of four iNOA classes, and found that the apoptotic capacity of germ cells in patients with iNOA was significantly higher than that in the normal group (Figure S2A, Supporting Information). Moreover, genes related to gamete generation, and energy production were downregulated in all classes except for Class II (Figure 2C and Figure S2A; Table S3, Supporting Information).

Based on the GO terms, we evaluated the cell cycle status in the four classes of iNOA by assessing the expression levels of G1/S and G2/M phase genes. In general, the overall expression level of cell cycle related genes in iNOA group was higher than that in NC group (Figure 2D; Table S3, Supporting Information). The ratio of cells with cell cycle activity to total cells was significantly higher in all four classes of iNOA than in the NC (Figure 2E). Further validation demonstrated that cell cycle marker KI67^[^ ^29^ ^]^ positive cells increased in iNOA, which was consistent with the GO analysis results shown in Figure 2B, suggesting that germ cells were more capable of cell cycle activity in iNOA patients (Figure 2F).

We next conducted pseudotime analysis of all germ cells in the four classes iNOA to assess whether there were developmental problems in the iNOA patients. We combined the germ cells of the iNOA and NC groups to predict the developmental order. The results showed that, in general, the trajectories of all classes of iNOA were consistent with those of NC, except for Class II, which had a small bifurcation (Figure S2B, Supporting Information). To exclude the influence of cells from NC on the developmental trajectory, we separately analyzed the developmental sequence of all germ cells in each class of iNOA. The results were similar to those for the combined results, suggesting that in the iNOA state, the cell fate of germ cells remains unchanged, except for a slight deviation in the timing of development (Figure S2C, Supporting Information).

### 2.3 Candidate iNOA Pathogenic Genes and Their Functions

**Figure 3:**
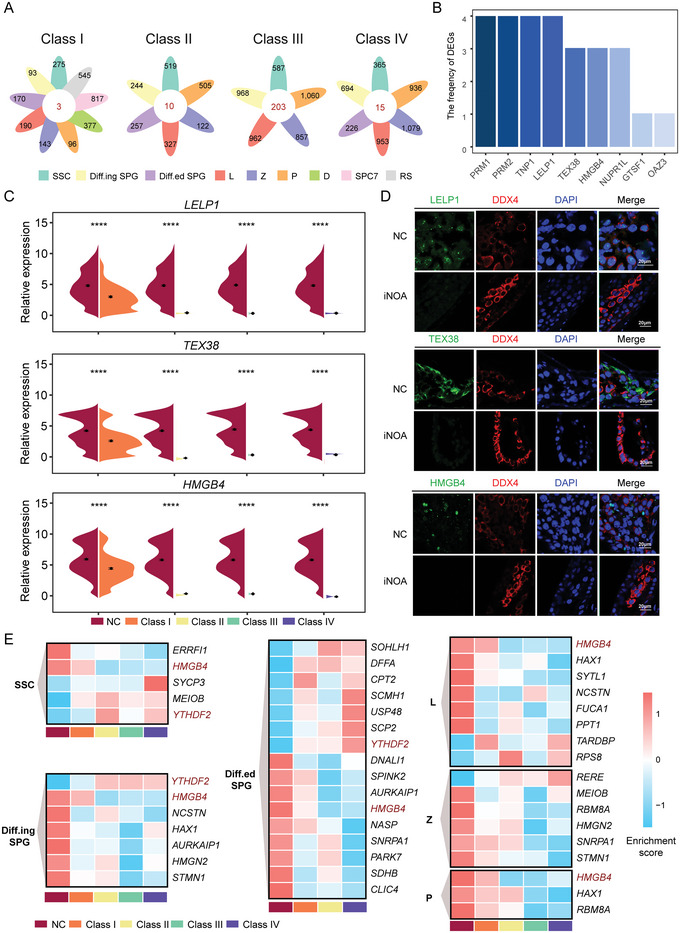
Candidate pathogenic genes for four classes idiopathic nonobstructive azoospermia (iNOA). A) Numbers of cell type specific downregulated genes in each iNOA class compared with normal controls (NC). B) High‐frequency genes. The ordinate (frequency of differentially expressed genes (DEGs)) represents the number of occurrences in the four classes iNOA. For example, “3” represents the number of downregulated genes in all germ cell stages in the three classes iNOA. Round sperm (RS) cells were excluded in Class II‐IV. C) Violin diagram showing gene expression levels. The two‐tailed Mann–Whitney–Wilcoxon test was used to assess significance. RS cells were excluded in Class II‐IV. D) Immunofluorescence of DDX4 (red) costaining of the target proteins (green) in NC and iNOA. The scale bar represents 20 µm. E) Expression patterns of Online Mendelian Inheritance in Man (OMIM) genes in each cell type. High and low enrichment are indicated in red and blue, respectively.

Based on the transcriptional characteristics of iNOA of the four classes, we set out to identify genes with significantly altered expression levels when compared with NC samples, as these may be pathogenic genes. We first screened DEGs between NC and iNOA of the same cell type. Then, we identified DEGs that were present in all germ cells of each iNOA class and counted the frequencies of these genes in the four classes (**Figure** **3**A). Among them, *PRM1*, *PRM2*, *TNP1*, and *LELP1* were DEGs in all four classes of iNOA. *TEX38*, *HMGB4*, and *NUPR1L* were downregulated in the three classes. *GTSF1* and *OAZ3* were found in one class (Figure 3B). The expression levels of these genes were significantly lower in germ cells of all stages in iNOA than NC (Figure 3C and Figures S3A,S3B,S4A,S4B, Supporting Information). To verify the reliability of the control data, we also compared transcriptome data from multiple studies (Figure S3C, Supporting Information). *PRM1*, *PRM2*, and *TNP1* encode protamine and transition proteins, which play important roles in nuclear remodeling and condensation during spermiogenesis.^[^ ^30^ ^]^ *PRM1* and *PRM2* polymorphisms have been associated with an elevated risk of male infertility, indirectly indicating the accuracy of our results.^[^ ^31^ ^]^ Immunostaining was performed to validate the expression patterns of these genes. The protein expression of these genes was significantly lower in iNOA samples than in NC samples (Figure 3D and Figure S4C, Supporting Information).

Online Mendelian Inheritance in Man (OMIM) is a comprehensive and authoritative database of associated human genotypes and phenotypes. We screened out all genes related to male infertility, azoospermia, and testicular tumor phenotypes from the OMIM database to further examine the expression patterns of these genes in our single‐cell data. *HMGB4* (high mobility group box 4) was downregulated in nearly all spermatogonia and spermatocyte stages in the iNOA samples. *YTHDF2* (YTH N6‐methyladenosine RNA binding protein 2), the first m6A reader discovered,^[^ ^32^ ^]^ was upregulated in all spermatogonia stages in the iNOA samples (Figure 3E). Thus, these genes are potential candidates for the development of NOA, and their functions in spermatogenesis should be further explored.

### 2.4 *CD164* is a Crucial Factor Regulating Spermatogonia Development

**Figure 4:**
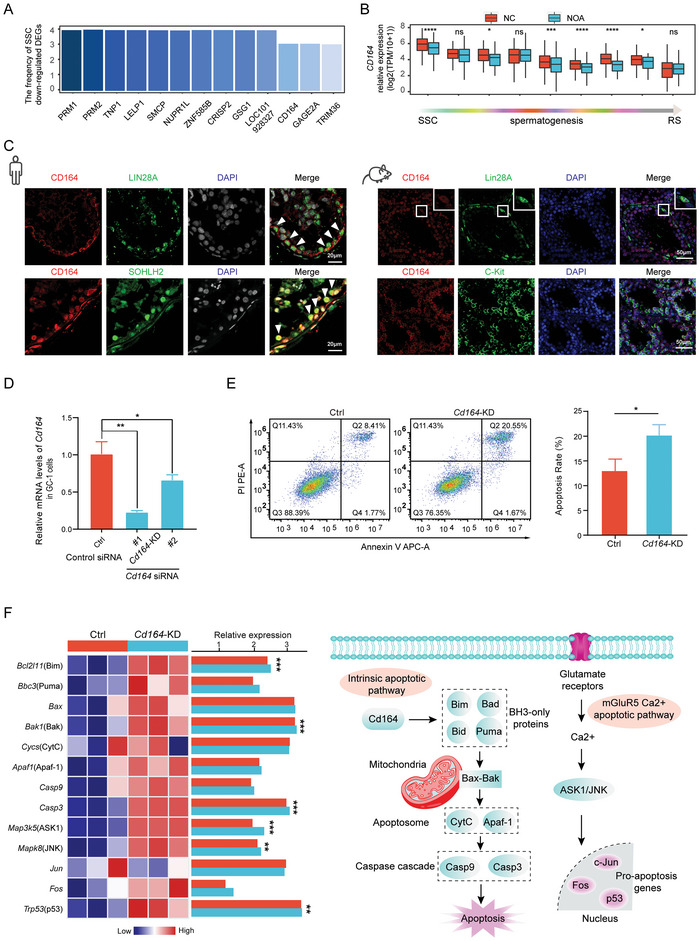
*CD164* plays an important role in the apoptosis of spermatogonia. A) High‐frequency genes downregulated in spermatogonial stem cells (SSC) of four classes idiopathic nonobstructive azoospermia (iNOA). B) The expression levels of *CD164* in human spermatogenesis. The two‐tailed Mann–Whitney–Wilcoxon test was used to assess significance. C) Immunostaining of CD164, LIN28A (a marker for undifferentiated spermatogonia), and SOHLH2 (a marker for differentiated spermatogonia) in testis sections from human adults. The DNA was stained with DAPI. Scale bar = 20 µm. Immunostaining of CD164, Lin28A (a marker for undifferentiated spermatogonia) and C‐Kit (a marker for differentiated spermatogonial) in testis sections from wild type mice at the indicated times. The DNA was stained with DAPI. Scale bar = 50 µm. D) *Cd164* mRNA levels in GC1 cells infected with either two independent *Cd164* siRNA or a scrambled siRNA (Ctrl siRNA). The results are normalized to the *Gapdh* mRNA level. *n* = 3, Error bars, S.D. ^*^p < 0.05, ^**^p < 0.01 by two‐tailed Student's *t* test. E) GC1 cells stained with Annexin V‐FITC/PI were analyzed by flow cytometry, Q1: dead cells (FITC^−^/PI^+^), Q2: late apoptosis (FITC^+^/PI^+^), Q3: viable cells (FITC^−^/PI^−^), Q4: early apoptosis (FITC^+^/PI^−^). Histogram of the total apoptotic rate in different groups. The total apoptosis rate was defined as late apoptosis plus early apoptosis. *n* = 3, Error bars, S.D. ^*^ *p* < 0.05 by two‐tailed Student's *t* test. F) Gene expression patterns of molecules in the apoptosis signaling pathway and the possible mechanism by which *CD164* promotes spermatogonia apoptosis. The two‐tailed Student's *t* test was used to assess significance.

Spermatogonial stem cells (SSC) in the testis provide the basis for spermatogenesis throughout life, and the spermatogenesis process is likely to be disrupted due to disorders in SSC.^[^ ^33^ ^]^ Therefore, we first concentrated on the DEGs in SSC of NC and iNOA, and found that several genes were significantly downregulated in multiple types of iNOA patients, including *PRM1*, *PRM2*, *TNP1*, *LELP1*, *SMCP*, and *CD164* (**Figure** **4**A,B). *CD164* (also known as endolyn) encodes a transmembrane sialic acid and cell adhesion molecule that interacts with C‐X‐C chemokine receptor type 4 to regulate muscle development,^[^ ^34^ ^]^ but its function in spermatogenesis has not been reported. Immunostaining showed that CD164 colocalized with undifferentiated (LIN28A^+^) and differentiated spermatogonia (SOHLH2^+^) in testes of human adults. In mice, the localization of CD164 was consistent with that in human, it colocalized with undifferentiated (Lin28A^+^) and differentiated spermatogonia (C‐Kit^+^) during mouse spermatogenesis (Figure 4C). This suggested that CD164 might play an important regulatory role in spermatogonia development, but the specific mechanism remains to be elucidated.

To further investigate the association between CD164 expression and spermatogonia development, the mouse spermatogonia derived GC‐1 spg (GC‐1) cell line was used as a model for in vitro functional assays. First, GC‐1 cells were transfected with *Cd164* siRNA (*Cd164*‐KD) and *Cd164* mRNA was successfully knocked down (Figure 4D). The rate of apoptosis was 20.55 ± 1.67% in GC1 cells with *Cd164* downregulation, which was significantly higher than that in the negative controls (Ctrl) (8.41 ± 1.77%; *p* < 0.05) (Figure 4E). These data demonstrated that *Cd164* contributed to spermatogonia apoptosis.

To investigate the mechanism of *Cd164*, transcriptomic studies were performed in the *Cd164*‐KD and Ctrl groups. Knockdown of *Cd164* resulted in activation of genes involved in cell chemotaxis and migration, and inhibition of differentiation genes (Figure S4D, Supporting Information). Moreover, the expression levels of *Bim*, *Bak*, *Casp9*, ASK1/JNK, and *p53* were significantly elevated following *Cd164* knockdown. These data indicated that *Cd164* knockdown activated the intrinsic apoptotic and mGluR5 Ca^2+^ apoptotic signaling pathways, which may result in spermatogonia apoptosis (Figure 4F).

### 2.5 Multilineage Interactions in Seminiferous Tubules and Their Dynamic Changes Under Disease Conditions

We delineated the molecular differences between iNOA and healthy subjects with normal spermatogenesis. Complex multilineage interactions in seminiferous tubules play an important role in the progression of azoospermia. Here, we used CellPhoneDB,^[^ ^35^ ^]^ a well‐established method for the inference of intercellular communication, to predict cell–cell interactions in the iNOA samples, as well as the dynamic changes from the normal samples.

**Figure 5:**
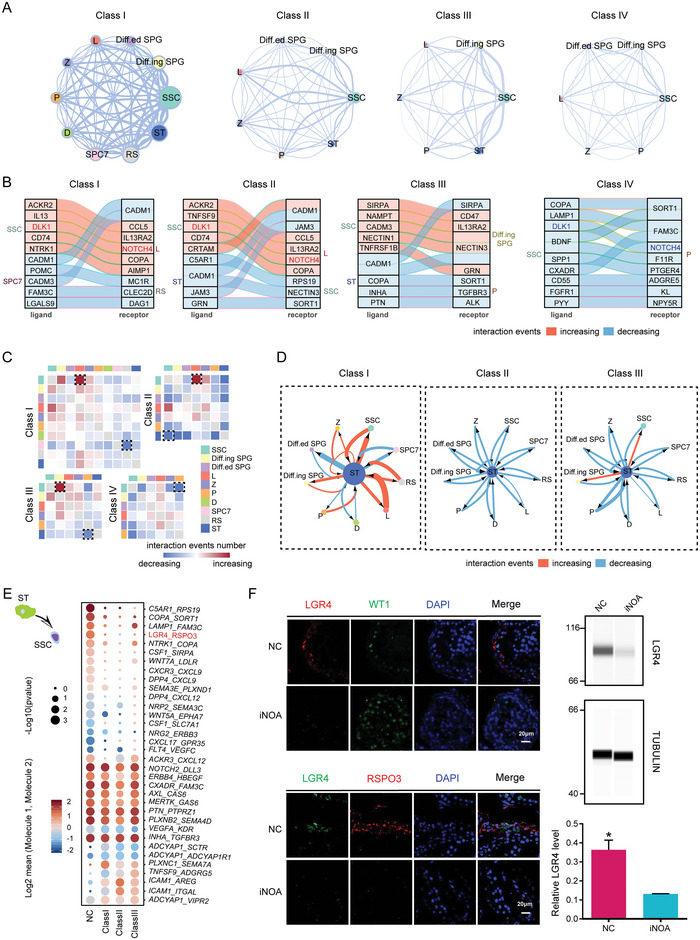
Multilineage interactions in seminiferous tubules. A) Network plots showing ligand–receptor interaction events between different cell types in the four idiopathic nonobstructive azoospermia (iNOA) classes. Cell–cell interactions are indicated by the connecting lines. Line thickness is proportionate with the number of interaction events. Node size represents interaction strength. B) Top 10 ligand–receptor pairs of cell types with the most variation in the number of interactions. C) Heatmaps showing the changes in ligand–receptor interaction events in each class of iNOA compared to the normal controls (NC) group. Red and blue indicate an increase or decrease, respectively, in the number of interactions compared to the NC group. D) Network plots showing the changes in ligand–receptor interaction events between Sertoli cells (ST) and germ cells in each class of iNOA compared to the NC group. Cell–cell interactions are indicated by the connecting lines. Line thickness is proportionate with the number of interaction events. Arrows indicate the direction of the interaction. Red and blue indicate an increase or decrease, respectively, in the number of interactions compared to the NC group. (E) Interaction of ST with spermatogonial stem cells (SSC). Ligands (front) were expressed on ST and receptors (back) were expressed on SSC. Dot size represents significance, defined as −log_10_ (*p*‐value). Color bars from blue to red represent the normalized expression values of both ligands and receptors. F) Immunofluorescence of LGR4, RSPO3, and the Sertoli cell marker WT1 in NC and iNOA. The scale bar represents 20 µm. Immunoblotting of LGR4 in testicular tissue of NC and iNOA. Quantification of protein levels is shown in the bottom section. Three samples from each group were analyzed. The two‐tailed Student's t test was used for significance test.

Interestingly, the analysis revealed hundreds of significant interactions (Table S4, Supporting Information). In particular, SSC were the most active cells, with the largest number of interaction events, among all cell types in the four classes of iNOA, suggesting that SSC act as hubs in the cell–cell interaction network during spermatogenesis (**Figure** **5**A). Next, we compared the interactions with those in NC. We found that Notch signaling, which is implicated in cell fate decisions and multiple developmental processes, was enhanced in Class I and II SSC, but attenuated in Class IV SSC (Figure 5B). Overall, Class II iNOA had the most numerous interaction changes, implying that this class differs the most from normal spermatogenesis. The interaction of SSC changed the most in all classes (Figure 5C).

The development from spermatogonia to spermatozoa in seminiferous tubules is supported by close intercellular communication between germ cells and ST.^[^ ^36^ ^]^ Our analysis showed that the interactions between ST and germ cells were mostly enhanced, whereas a small fraction was weakened in Class I when compared with the NC samples, and interactions in Class II and Class III were nearly all attenuated (Figure 5D). We further analyzed interacting pairs of ligands on ST and receptors on SSC and found that the expression of the *LGR4*/*RSPO3* interaction pair was significantly reduced in iNOA samples (Figures 5E,F). In *LGR4* knockout mice, male reproductive tract dysplasia occurs after birth.^[^ ^37^ ^]^ Conversely, we focused on the interaction of SSC with ST, and found that the *CD226*/*NECTIN2* and *NOTCH1*/*JAG2* interaction pairs were downregulated in all classes of iNOA (Figure S5A, S5B, Supporting Information). It has been reported that spermatogenesis in *Nectin2* knockout mice deviates from the normal developmental process, resulting in an infertile phenotype.^[^ ^38^, ^39^ ^]^ Decreased activity of the Notch1 and Jagged 2 system may lead to cell maturation arrest in human testes.^[^ ^40^ ^]^ We also analyzed the interactions between ST and other cells, and the results revealed that there were dynamic changes in the iNOA state (Figure S5C–E, Supporting Information).

### 2.6 Reconstruction of Transcriptional Regulatory Networks in iNOA

To better understand the dynamics of transcriptional regulatory networks in iNOA patients, we predicted core TFs that may regulate male infertility in the four classes of iNOA (Table S5, Supporting Information). We first pooled the data of all germ cells to find the common TFs in all classes of iNOA. A total of 27 TFs with decreased transcriptional activity and 66 TFs with increased transcriptional activity were found in the iNOA samples (Figure S6A, Supporting Information). Among them, nine TFs showed no difference in expression between NC and iNOA. In addition, there were three TFs with a negative correlation between transcriptional activity and the expression level (Figure S6B, Supporting Information). Next, we performed GO analysis of the two groups of TFs with differential transcriptional activity to explore the functions of common TFs in iNOA. Interestingly, the TFs with decreased transcriptional activity were involved in determination of left/right symmetry, cell maturation, and spermatogenesis. This is consistent with recent findings that ST in NOA patients tend to be immature.^[^ ^24^ ^]^ Moreover, TFs that regulate covalent chromatin modification and cell cycle were more active, in line with the results shown in Figure 2 and Figure S6C (Supporting Information). We found three TFs and their target genes related to spermatogenesis, including *CREM*, *HSF1*, and *RFX2*, which are involved in cell maturation, spermatogenesis, and energy metabolism (Figure S6D, Supporting Information).

To identify cell type‐specific TFs, we screened TFs with differential transcriptional activity in the same cell type between the iNOA and NC samples. There were a few TFs with altered transcriptional activity in Classes I and II, but numerous TFs with altered transcriptional activity in Classes III and IV (Figure S7A, Supporting Information). These TFs with altered transcriptional activity were rarely present in the four classes of iNOA in the early stage of spermatogenesis, but appeared in the late stage, indicating that various types of azoospermia have different features in the early stage, but numerous common features in the late stage (Figure S7B, Supporting Information). To identify TFs that were dysregulated in all stages, we determined the frequency of cell‐specific TFs in the four classes of iNOA. Surprisingly, we found increased transcriptional activity of *HES7* in iNOA cells at multiple stages, but decreased transcriptional activity of *SOX5* at all stages (Figure S7C, Supporting Information). Target genes of *SOX5* in Class I were mainly related to peptidyl‐tyrosine dephosphorylation involved in inactivation of protein kinase activity and the sphingolipid signaling pathway, whereas target genes in Class III were mainly involved in the Wnt signaling pathway and spermatogenesis‐related pathway (Figure S7D, Supporting Information).

### 2.7 Identification of Genes Predicting Testicular Spermatogenic Capacity

**Figure 6:**
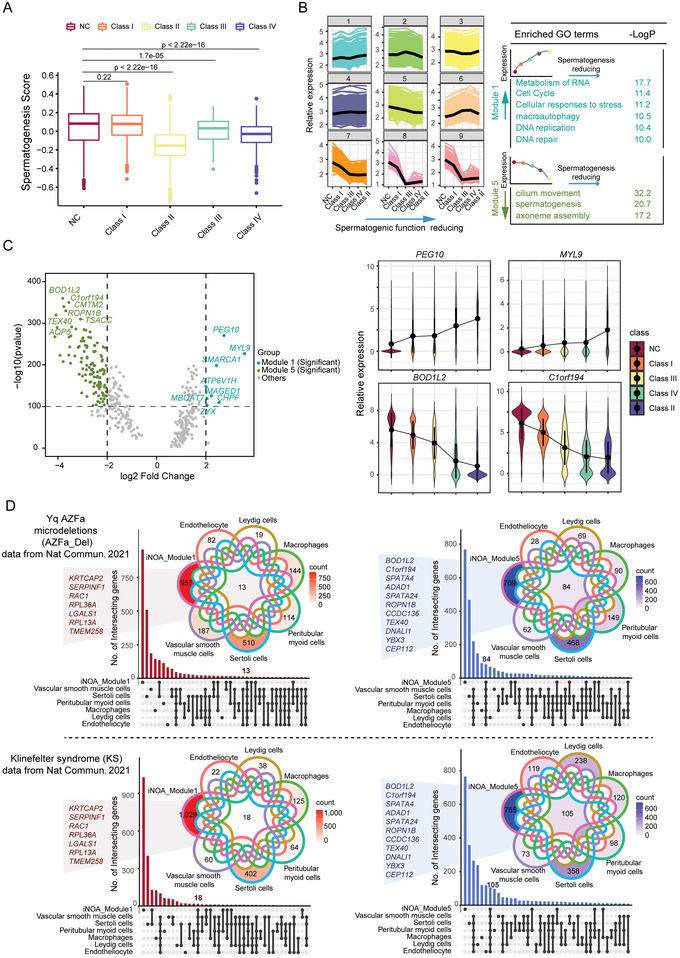
Identification of genes predicting testicular spermatogenic capacity. A) Spermatogenesis scores of each type of idiopathic nonobstructive azoospermia (iNOA). The two‐tailed Mann–Whitney–Wilcoxon test was used to assess significance. B) Ranking of normal controls (NC) and the four classes iNOA based on spermatogenesis scores. Trend genes related to ranking and associated GO terms. Module 1 includes genes whose expression increased with reducing spermatogenic capacity; Module 5 includes genes whose expression decreased with reducing spermatogenic capacity. C) Volcano plot showing the most significantly up/downregulated genes with testicular spermatogenic capacity. Line plot showing gene expression trends. D) Comparison of iNOA trend genes and differentially expressed genes (DEGs) in other testicular diseases, such as Y‐chromosome microdeletions and Klinefelter syndrome. On the left is the Module 1 gene compared to DEGs that are upregulated in testicular diseases; on the right is a comparison of the Module 5 gene with DEGs downregulated in testicular diseases. The *X* axis represents the intersection of module genes and DEGs in testicular diseases, compared to NC. The *Y* axis is the number of intersecting genes.

To identify the subtypes and arrest stages of azoospermia and help clinicians better diagnose it, we set out to identify genes that are indicative of testicular spermatogenic capacity. We first calculated the spermatogenesis scores of each type in iNOA, based on the expression levels of spermatogenesis‐related genes (Experimental Section). The spermatogenesis scores were in the order NC group > Class I > Class III > Class IV > Class II, correlating with low to high severity of azoospermia, which is consistent with the results shown in Figure 1 (**Figure** **6**A). Then, we assessed the gene expression trends and found that, the weaker the spermatogenesis capacity in the testis, the higher the expression levels of the genes in Module 1, and the lower the expression levels of the genes in Module 5. GO analysis and correlation analysis showed that genes negatively correlated with spermatogenesis capacity were mainly related to cell cycle and cellular responses to stress and DNA repair, whereas genes positively correlated with spermatogenesis capacity were mainly associated with cilium movement and spermatogenesis (Figure 6B and Figure S8A,S8B, Supporting Information). *PEG10*, *MYL9*, *BOD1L2*, and *C1orf194* showed the most obvious alterations (Figure 6C). *PEG10*, an imprinted gene essential for embryo development and placentation, is reportedly hypermethylated in spermatozoa of the male partners in recurrent miscarriage couples.^[^ ^41^ ^]^ *BOD1L2* has been reported to play an important role in detecting or correcting the attachment of mitotic spindles in the biorientation of chromosomes.^[^ ^42^ ^]^

To test the diagnostic power of the genes we identified as testicular spermatogenic ability predictors in other testicular diseases, we reanalyzed published single cell RNA‐seq data for testicular diseases.^[^ ^43^, ^44^ ^]^ Our results showed that testicular spermatogenic ability predictors may also play a role in the diagnosis of spermatogenesis in Y chromosome microdeletions, Klinefelter syndrome (KS), and cryptozoospermia. In particular, the most significantly altered trend genes, such as *BOD1L2* and *C1orf194*, were prominent (Figure 6D and Figure S8C, Supporting Information). These data suggested that testicular spermatogenic ability predictors had the potential to diagnose spermatogenesis in a variety of testicular diseases and could be promising markers for clinical diagnosis in the future.

## 3 Discussion

We performed scRNA‐seq analysis of 3696 individual testicular cells from 17 iNOA donors to construct transcriptional landscapes of human spermatogenesis in the context of NOA. In comparison with healthy subjects with normal spermatogenesis, we assessed the gene expression characteristics of germ cells and somatic cells, cell–cell interactions between germ cells and somatic cells, as well as the regulatory networks of TFs in iNOA patients. In particular, we demonstrated that *CD164* played an important role in the apoptosis of spermatogonia. Moreover, we identified a series of genes predicting spermatogenic capacity in different testicular diseases. In general, our study provides new insights into the molecular pathogenesis as well as the clinical diagnosis and therapeutic strategy of iNOA.

Our results revealed that germ cells, including SSC, from azoospermia patients had enhanced cell cycle activity and weakened energy production, which may provide clues for the selection of factors to be added to the in vitro culture medium for round sperm. As SSC can self‐renew to maintain the stem cell population and further produce sperm, sperm can be produced uninterruptedly throughout adulthood.

To identify candidate iNOA pathogenic genes, we analyzed the key DEGs between the four classes iNOA and the NC group. Among them, *HMGB4*, a member of the high‐mobility group box (HMGB) family, is present in spermatocytes, spermatids, and spermatozoa in human and mice.^[^ ^45^, ^46^ ^]^ A significant decline of *HMGB4* in all cell types in the three classes of iNOA was observed. Though male fertility was not affected in *Hmgb4* knockout mice,^[^ ^47^ ^]^ which might be due to species variation. We speculated that HMGB4 might be a key pathogenic gene of iNOA and played an important role in spermatogenesis. More studies should be carried out in the future. Cell–cell interactions between ST and developing SSC have long received research interest. We found that the CD226/NECTIN2, LGR4/RSPO3, and NOTCH1/JAG2 interaction pairs might have an effect on human spermatogenesis. Moreover, the CSF1/SIRPA and CXCL12/CXCR4 interaction pairs were downregulated in all classes of iNOA. CSF1 (colony‐stimulating factor 1) exerts a crucial impact on the development of SSC.^[^ ^48^ ^]^ Polymorphisms in *SIRPA*, its interaction partner, are strongly associated with iNOA susceptibility.^[^ ^49^ ^]^ Additionally, *CXCL12* encodes a chemokine expressed and secreted by ST and binds to the CXCR4 receptor on SSC to regulate the self‐renewal and maintenance of SSC.^[^ ^50^, ^51^ ^]^ Therefore, the downregulation of these key interaction signals may provide ideas for targeted therapy of iNOA in the future.

Apart from intercellular communication, TFs are essential in spermatogenesis. We found that HES7 (Hes family bHLH TF 7), as a transcriptional repressor,^[^ ^52^ ^]^ was significantly upregulated in iNOA cells. Another key TF, SOX5, is necessary for correct gene expression patterns during spermatogenesis^[^ ^53^ ^]^ and its expression was significantly downregulated in iNOA cells. Therefore, we speculate that the increase in *HES7* transcriptional activity and decrease in *SOX5* transcriptional activity may disrupt the key regulatory network during spermatogenesis and cause iNOA.

None of the iNOA patients in our study had mature sperm, but other indicators, such as genetic factors, were normal in all patients. We classified them into four groups based on gene expression and pathological features, and identified a series of genes that were highly associated with spermatogenic capacity. At present, testicular biopsy is an invasive method to diagnose the spermatogenic ability of patients, and a safe and noninvasive diagnostic strategy is still lacking. The genes identified in this work may be integrated into a panel for the rapid and noninvasive diagnosis of male infertility in the future.

As demonstrated in this study, the interactions between germ cells and somatic cells were speculated only by expression data, computational software prediction, protein localization, and quantification. Additional experiments are needed to further clarify the cell–cell communications. In addition, we used cell lines for functional validation of the key pathogenic genes we screened, indicating the reliability of our bioinformatics analysis results. Further studies using in vivo animal models are still needed to better characterize the effect of these pathogenic genes on spermatogenesis. Moreover, we used different testicular diseases to find common diagnostic predictor of spermatogenic ability, providing a large amount of data resources for clinical diagnosis. However, a large number of clinical samples still need to be included to verify its accuracy.

Although there have been enormous advances in iNOA testicular microenvironment, transcriptional network of autophagy‐related genes, and hub genes of azoospermia,^[^ ^24^, ^54^, ^55^ ^]^ to our knowledge, this was the first study to investigate the occurrence and development of idiopathic NOA from a testicular germ cell perspective, including the largest number of cases to date. The data comprehensively and systematically reveal the transcriptional regulatory network of iNOA. Our findings not only provide valuable knowledge to improve our understanding of the molecular basis and mechanism of iNOA but also provide new insights for clinical diagnosis and treatment.

## 4 Experimental Section

### Experimental Model and Subject Details

The donors in this study underwent sperm isolation surgery for in vitro fertilization due to male‐related infertility. Men with spermatogenic cells, but without mature sperm, abnormal hormone levels, chromosomal abnormalities, Y chromosome microdeletion, and no other medical history were selected. Testis tissues were collected from 17 iNOA patients between 27 and 37 years old to map single‐cell transcriptome landscapes. All testis samples for transcriptome analysis were obtained from Peking University Third Hospital with informed consent from the donors. The study was approved by the Ethics Committee of Peking University Third Hospital (2017SZ‐048). All experimental procedures and animal care were approved by the Animal Care and Use Committee of the Peking University Health Science Center (LA2021579). Normal Samples data included 2854 testicular cells from 9 donors, have been deposited in NCBI GEO: GSE106487.

### Isolation of iNOA Male Testicular Cells for Transcriptional Profiling

Idiopathic NOA testis tissues were washed three times with Dulbecco's modified Eagle's medium (DMEM, C11995500BT, Thermo Scientific) containing 10% fetal bovine serum (FBS, 10437028, Gbico) and minced using sterilized scissors. Then, 500 µL of Accutase Cell Detachment Solution (A6964, Sigma‐Aldrich) was added, and the samples were incubated at 37 °C for 15 min. To completely digest the tissues into single cells, the mixture was pipetted 30 times at 7 min and at the end of digestion. The cell suspension was filtered through 40 µm Pre‐Separation Filters and centrifuged at 300× *g* for 8 min, and the cells were resuspended in 500 µL of DMEM (containing 10% FBS).

### ScRNA‐seq Library Preparation and Sequencing

After digestion, the cells were diluted in 1 mg mL^−1^ Ac‐Bovine Serum Albumin (A9418, Sigma‐Aldrich) and single cells were randomly picked using a mouth pipette and added to lysis buffer prepared in advance. Mouth pipette method can make sure that there's only one cell in each tube. A previously reported modified STRT‐seq protocol was used to construct the scRNA‐seq library.^[^ ^56^ ^]^ In brief, after total RNA was extracted from the single cells, the mRNAs were captured using an oligo (dT) primer and subjected to reverse transcription. The cDNAs were PCR‐amplified in 18 cycles to increase yields. After barcoding, the cDNAs from 48 single cells were pooled, and a biotin‐modified index sequence was added to the 3′ end in four cycles of PCR. Dynabeads MyOne Streptavidin C1(65 002, Invitrogen) was used to capture the fragmented 3′ cDNAs, which were sheared using Covaris (S2). The libraries were constructed using a Kapa Hyper Prep Kit (KK8505, Kapa Biosystems) and were subjected to 150 bp paired‐end sequencing on the Illumina 4000 platform.

### Bulk Full‐Length Transcriptome Library Preparation and Sequencing

RNA sample purity, concentration, and integrity were detected with NanoPhotometer spectrophotometer and Agilent 2100 RNA Nano 6000 Assay Kit (5067‐1511, Agilent Technologies, CA, USA). Then, 1–3 µg of total RNA was used as starting material to construct a transcriptome sequencing library for each sample. According to the operation instructions of VAHTS Universal V6 RNA‐Seq Library Prep Kit for Illumina (NR604‐01/02), different index tags were selected to construct the library. Then, mRNA was enriched by magnetic beads with Oligo (dT), which were further fragmented into fragments by fragmentation buffer. mRNA was used as the template to synthesize cDNA, and then double‐stranded cDNA was purified by AMPure P Beads or QiaQuick PCR kit. The purified double‐stranded cDNA was then end repaired. A tail was added, and the sequencing adaptor was connected. Then, the fragment size was selected, and the final cDNA library was obtained by PCR rich set. After quality control, the library was subjected to 150 bp paired‐end sequencing on a NovaSeq 6000 S4 platform.

### Processing of the Single Cell Transcriptome Data

First, the pooled raw paired‐end reads were separated based on the unique cell barcode in read 2. Second, the corresponding read 1 was obtained based on the read ID. Third, the TSO sequence, the tail sequence of PolyA, low‐quality bases (*N* > 10%), and adapter contamination in read 1 were pruned. Next, clean read 1 was aligned to the hg19 human reference genome (UCSC), using TopHat. Then, the uniquely mapped reads were counted, using the HTSeq package, and removed PCR biases based on UMI information. After that, the transcripts per gene were quantified and downstream analysis was performed using log_2_ (TPM/10 + 1). All ribosomal and mitochondrial genes were excluded in the downstream analysis. The low‐quality cells were filtered out to avoid affecting the results of subsequent analysis, and cells with more than 2000 genes and more than 10000 transcripts were only retained.

### Patient Classification and Cell Type Identification

To classify the 17 samples and find common characteristics, cluster analysis was performed on all cells according to patient using hclust in R. It was observed whether the cells distribution and pathological features of different patients were consistent to verify the correctness of the clustering results.

The four classes iNOA were separately compared with normal cells for cell type identification. The R package Seurat was used to normalize the TPM expression levels. To collect the same cell type for comparison with the normal data, the highly variable genes previously found in the normal data were used as input genes for subsequent principal component analysis. The significant principal components (PCs) were selected through jackstraw analysis with 100 replicates to perform dimension reduction analysis using the RunUMAP function. After obtaining the automatic clustering results, the cell type of each cell cluster was defined based on the expression of classical marker genes.

### Identification and Functional Enrichment of Differentially Expressed Genes in Each Cell Type

The FindMarkers function (test.use = “wilcox”, thresh.use = 2, min.pct = 0.25) were used in the Seurat package on normalized TPM expression levels to identify DEGs among the cell types. GO analysis was performed using Metascape (https://metascape.org/).

### Cell Cycle Analysis and Construction Development Trajectories of 4 Classes iNOA

For cell cycle analysis, the expression values of 43 G1/S and 54 G2/M cell cycle‐related genes were calculated to assess the cell cycle status in the four classes of iNOA (Table S3, Supporting Information). For pseudotime analysis, the intersection of highly variable genes previously found in the normal data and expressed genes as input genes were used in Monocle v.2.14.0 to construct the trajectory landscape of spermatogenesis in the four classes iNOA.

### Cell–Cell Interaction Analysis

The CellPhoneDB software (www.cellphonedb.org), which includes ligands, receptors, and interaction modules, was used to predict cell–cell communication. Only the receptors and ligands that were expressed in more than 10% of the specific cell types were further analyzed, and if the ligand or receptor was not detected, it was considered that there was no interaction. Comparing the average expression of each ligand–receptor pair between the different cell types, only *p* < 0.05 was considered to indicate reliable cell–cell communication.

### TF‐Target Regulatory Network Analysis

TF regulatory network analysis was performed using the pySCENIC workflow with default parameters (https://github.com/aertslab/pySCENIC). The process included: 1) inference of the regulators composed of TFs and target genes using correlations between the expression of all genes in cells, 2) refinement of the enriched regulators by pruning targets that have no corresponding motif, which can effectively distinguish direct targets from indirect targets based on the presence of cis‐regulatory footprints, and 3) calculation of the transcriptional activity of the regulators identified in each cell.

### Immunofluorescence Analysis of the Human and Mouse Testis

Human testicular biopsies were fixed in 4% paraformaldehyde solution for 2 h, embedded in optimal cutting temperature compound (OCT, 4583, Sakura) and frozen in liquid nitrogen. The OCT‐embedded biopsies were sliced into 10 µm thick sections. Immunofluorescence staining was performed according to a reported protocol.^[^ ^22^ ^]^ After three washes in phosphate‐buffered saline with 0.3% Triton (PBST), the sections were blocked in 5% bovine serum albumin (BSA, A1933, Sigma) in PBST at room temperature for 1 h followed by incubation with primary antibodies against DDX4 (1:500, ab27591, Abcam), KI67 (1:500, ab21700, Abcam), LELP1 (1:100, ab229947, Abcam), TEX38 (1:100, 24429‐1‐AP, Proteintech), LIN28A (1:600, ab46020, Abcam), SOHLH2 (1:100, NPB2‐20453, NovusBio), CD164 (1:200, GTX17625, GeneTex), GTSF1 (1:100, PA5‐58575, Thermo Fisher Scientific), HMGB4 (1:100, ab224500, Abcam), and OAZ3 (1:100, PA5‐65056, Thermo Fisher Scientific) at 4 °C overnight.

Testes extracted from wild type male mice were isolated immediately after cervical dislocation and fixed in 4% paraformaldehyde (G1101, Servicebio) overnight at 4 °C for immunostaining. After stepwise dehydration using an ethanol series, the samples were embedded in paraffin and sectioned using a Leica slicing machine (Leica, RM2235, Germany). After dewaxing and hydration, the sections were boiled in Tris‐EDTA (pH 8.0) for 15 min, gradually cooled down to room temperature, washed in PBST, blocked with 3% BSA for 1 h at room temperature, and then incubated with primary antibodies against CD164 (1:200, GTX17625, GeneTex), Lin28A (1:600, ab46020, abcam), C‐Kit (1:100, D13A2, CST) overnight at 4 °C.

After three washes in PBST, the sections were incubated with secondary antibody (1:1000, A‐11008, A‐11004, A‐32731, and A11005, Thermo Fisher Scientific) and DAPI (1:1000, ab285390, Abcam) to stain the nuclei at room temperature for 1 h, followed by three washes with PBST. Images were captured with a ZEISS LSM880 inverted confocal microscope. Ten to fifteen regions were randomly selected for counting the positively stained cells.

### Capillary Electrophoresis Immunoblotting

Proteins were extracted from frozen testicular tissues. Thirty micrograms of proteins were denatured in Fluorescent Master Mix (PS‐FL01‐8, Protein Simple) at 95 °C for 5 min. All samples were analyzed on a Protein Simple Wes automated capillary‐based electrophoresis instrument (Simple Western System) according to the instructions (SM‐W004, Protein Simple) with primary antibodies, including rabbit anti‐CD226 (1:50, 17842‐1‐AP, Proteintech), rabbit anti‐LGR4 (1:50, PA5‐109908, Thermo Fisher), and mouse anti‐*α*‐tubulin (1:100, AC012, ABclonal). The data were quantitatively analyzed using Compass software (Compass Software version 5.0.1, Build ID 1258), and the protein signal strength (area) was normalized to control the peak area of *α*‐tubulin by loading.

### Cell Culture and siRNA Transfection

A mouse spermatogonium‐derived GC‐1spg (GC‐1) cell line^[^ ^57^ ^]^ was used as a cell culture model that has been authenticated to be functional for biochemical studies.^[^ ^58^, ^59^ ^]^ The GC‐1 cell line was obtained from the American Type Culture Collection (ATCC) and grown in DMEM (Thermo Scientific) supplemented with 10% FBS (Gibco) and 1% Penicillin–Streptomycin at 37 °C under 5% CO_2_. Cell transfection was performed using Lipofectamine RNAiMAX Transfection Reagent (13778150, Thermo Fisher) according to the manufacturer's instructions. In general, 100 nmol of *Cd164*‐siRNA (siRNA‐#1: GGACAACAAATACCACACT and siRNA‐#2: TCTGTAATACCTCCTACCA, hereafter referred to as *Cd164*‐KD) and the corresponding negative control (Ctrl) synthesized by RiboBio (Guangzhou, China) were used for each transfection in a 6‐well plate.

### RNA Extraction and qRT‐PCR

Total RNA was extracted from GC‐1 spg cell line, using TRIzol reagent (15596018, Thermo Scientific). The isolated RNA (0.5 µg) was reverse transcribed into cDNA using a PrimeScript Kit with gDNA Eraser (RR047A, TaKaRa). The mRNA levels were detected by qRT‐PCR with a SYBR premix reagent (A25742, Invitrogen) on an ABI QuantStudio 3 (Applied Biosystems). The results were calculated by the ΔΔCt quantification method, with *Gapdh* mRNA serving as an internal reference. Each experiment was performed independently at least three times. The validity of the qRT‐PCR data was assured by following the MIQE guidelines.^[^ ^60^ ^]^ Primer for *Gapdh*, Forward Primer: AGGTCGGTGTGAACGGATTTG, Reverse Primer: GGGGTCGTTGATGGCAACA. Primer for *Cd164*, Forward Primer: AGAAACCTGTGCGAGCTTCAA, Reverse Primer: CACAAGTCAGTGCGGTTCAC.

### Apoptosis Assay

GC‐1spg cell line was seeded in 6‐well plates and incubated with *Cd164*‐siRNA or Ctrl‐siRNA loaded for 48 h. After treatment, cells in 6‐well plates were washed with PBS, followed by binding buffer from an apoptosis analysis kit (AD11, DOJINDO). Then, 5 µL of FITC‐Annexin V and 5 µL of propidium iodide (PI) were added to the wells for 15 min of staining. To analyze the rate of apoptosis, all of the adherent and floating cells were collected and gently washed twice with PBS before resuspending them in 1 mL of binding buffer. After incubating with FITC‐Annexin V and PI in test tubes for 15 min at room temperature, the cells were subjected to flow cytometry analysis with Cytoflex S (Beckman Coulter, CA, USA), and the data was analyzed with CytExpert Software. The early stage apoptotic cells were marked with blue fluorescence, while the late‐stage apoptotic cells were labeled with both blue and red fluorescence.

### Spermatogenesis Score Calculation

All the genes associated with spermatogenesis were downloaded from the Gene Ontology database and the gene list was attached in Table S6 (Supporting Information). Then the *Z*‐score of the gene expression level in the spermatogenic cells was calculated. And the average *Z*‐score of all the genes was defined as the spermatogenesis score.

### Statistical Analysis

Relative expression levels were defined as log_2_ (TPM/10 + 1). R software was used for statistical analysis. The chi‐square test was used in Figure 1D. A two‐tailed Student's *t* test was used in Figures 2F, 4D–F, 5F and Figure S5B (Supporting Information). A two‐tailed Mann–Whitney–Wilcoxon test was used in Figure 3C, 4B and Figures S3,S4A,S4B (Supporting Information), Figure 6A. Statistically significant comparisons were shown, with significance defined as ^*^ *p* < 0.05, ^**^ *p* < 0.01, ^***^ *p* < 0.001. The sample size for statistical analysis was described in the legend of corresponding figure.

### Data and Software Availability

Data have been deposited in the National Genomics Data Center of the China National Center for Bioinformation (https://ngdc.cncb.ac.cn/gsa‐human/), with accession number HRA001477. Code for revevant analysis are available at https://github.com/jasminexiao/NOA‐related‐code. Further information should be directed to and will be fulfilled by the Lead Contact, Jie Qiao (jie.qiao@263.net).

## Conflict of Interest

The authors declare no conflict of interest.

## Author Contributions

Y.C., X.L., L.Z., and F.Z. contributed equally to this work. This project was conceived and coordinated by J.Q., H.J., and Q.L. Y.C. conducted all the studies and performed the bioinformatics analysis. X.L. carried out the sequencing experiments. L.Z. collected samples and performed the immunostaining with the help of L.Y, Z.Z, and W.T. And F.Z. completed the functional experiments of the cell lines. Y.C., X.L., L.Z., and F.Z. wrote the manuscript with contributions from all of the authors.

## Supporting information

Supporting Information

Supplemental Table 1

Supplemental Table 2

Supplemental Table 3

Supplemental Table 4

Supplemental Table 5

Supplemental Table 6

## Data Availability

Our data have been deposited in the National Genomics Data Center of the China National Center for Bioinformation (https://ngdc.cncb.ac.cn/gsa‐human/), with accession number HRA001477. Further information should be directed to and will be fulfilled by the Lead Contact, Jie Qiao (jie.qiao@263.net).
