# Supplementary material for: Deciphering the Molecular Characteristics of Human Idiopathic Nonobstructive Azoospermia from the Perspective of Germ Cells
Source: Adv Sci (Weinh). 2023 Apr 21;10(17):2206852. doi: 10.1002/advs.202206852 (PMC10265083; doi:10.1002/advs.202206852)
Supplement: Supplementary file 1 — Supporting Information [file ADVS-10-2206852-s001.pdf]

ADVANCED SCIENCE
Open Access

Supporting Information

for *Adv. Sci.*, DOI 10.1002/advs.202206852

Deciphering the Molecular Characteristics of Human Idiopathic Nonobstructive Azoospermia from the Perspective of Germ Cells

Yidong Chen, Xixi Liu, Li Zhang, Feiyin Zhu, Liying Yan, Wenhao Tang, Zhe Zhang, Qiang Liu, Hui Jiang* and Jie Qiao**

Supporting Information

Deciphering the molecular characteristics of human idiopathic non-obstructive azoospermia from the perspective of germ cells

Yidong Chen[1, 2, 3, 8], Xixi Liu[1, 2, 3, 8], Li Zhang[1, 2, 3, 8], Feiyin Zhu[1, 2, 3, 6, 8], Liying Yan[1, 2, 3], Wenhao Tang[1, 2, 3], Zhe Zhang[7], Qiang Liu[1, 2, 3, *], Hui Jiang[7, *] and Jie Qiao[1, 2, 3, 4, 5, 6, *]

Fig. S1

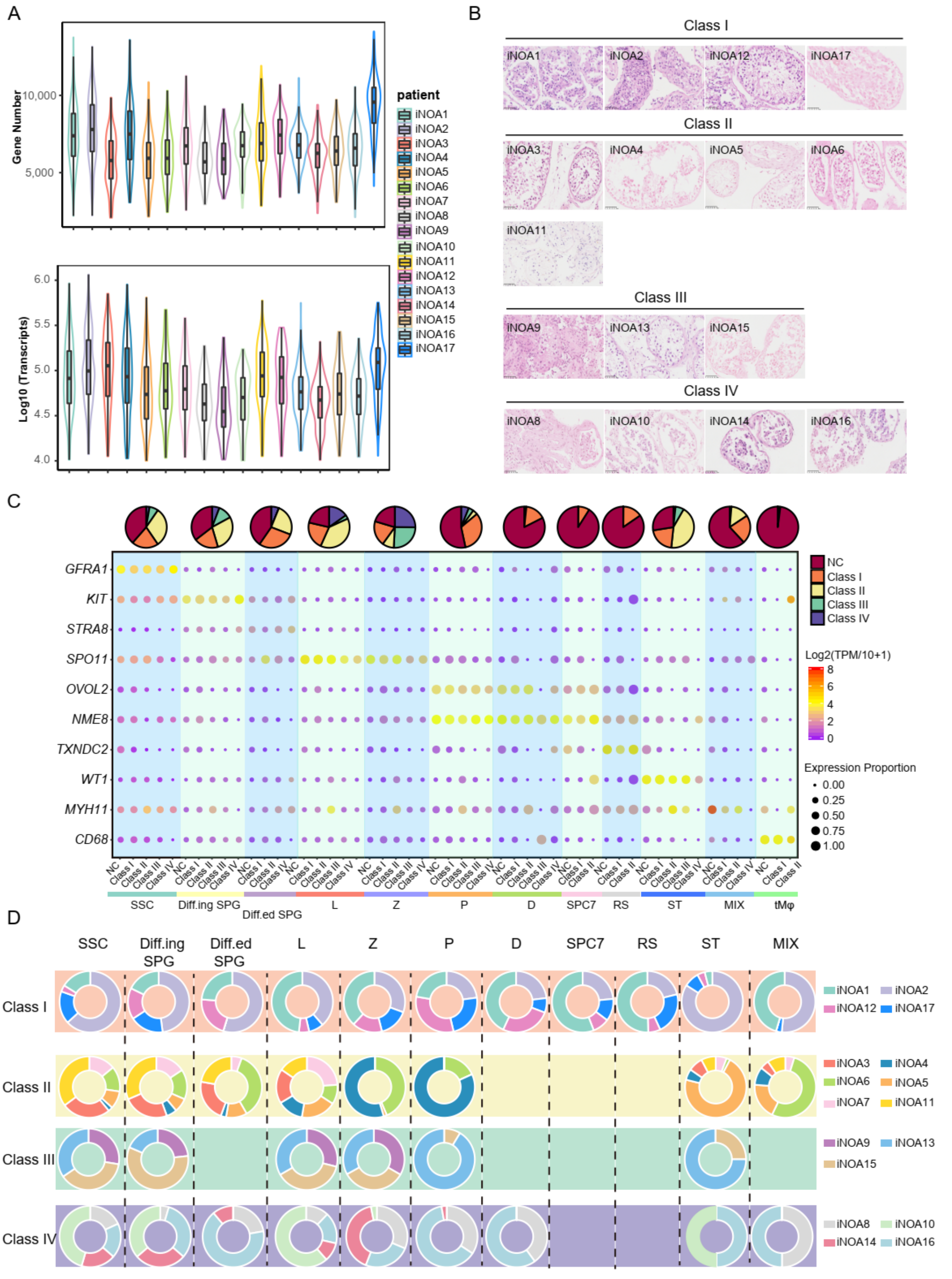

Figure S1. Quality control of RNA-seq data and cell composition in the four classes of iNOA. (**A**) Boxplots of the numbers of genes (up) and transcripts (down) detected in each single cell. (**B**) Immunohistochemistry of testicular tissues in iNOA patients. (**C**) Classic marker gene expression levels in NC and four classes of iNOA. SSC, spermatogonial stem cells; Diff.ing SPG, differentiating spermatogonia; Diff.ed

SPG, differentiated spermatogonia; L, leptotene spermatocytes; Z, zygotene spermatocytes; P, pachytene spermatocytes; D, diplotene spermatocytes; SPC7, spermatocyte 7; RS, round sperm; ST, Sertoli cells; MIX, mixture of peritubular myoid cells and Leydig cells; tMφ, testicular macrophages. *GFRA1*, marker of SSC; *KIT*, marker of Diff.ing SPG; *STRA8*, marker of Diff.ed SPG; *SPO11*, marker of meiosis; *OVOL2*, localized in the XY body of human spermatocytes at the pachytene and diplotene stages; *NME8*, expressed from pachytene spermatocytes to the early stage of spermatids; *TXNDC2*, marker of RS and elongated spermatids; *WT1*, marker of ST; *MYH11*, marker of peritubular myoid cells; *CD68*, marker of tMφ. (**D**) Cell source and ratio in each type of testicular cell in the four classes of iNOA.

Fig. S2

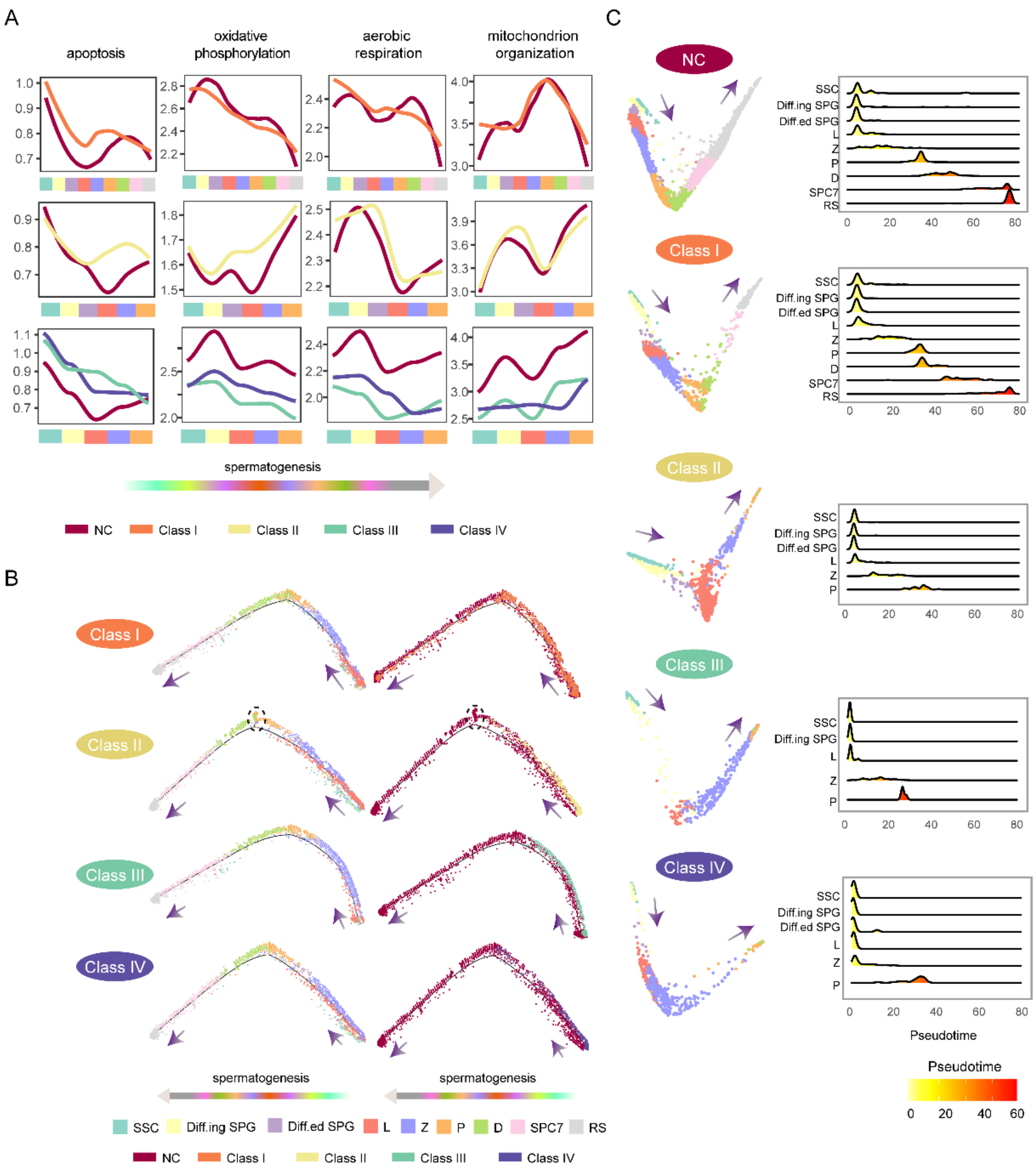

Figure S2. Pseudotime trajectory analysis of the iNOA Samples. (**A**) Expression levels of specific genes. (**B**) Developmental pseudotime of adult human male germ cells from the NC group and each iNOA class. Arrows indicate the developmental order of germ cells. The left column shows the pseudotime trajectory of each cell type,

and the right column shows the pseudotime trajectory of the cell origin. (**C**) Developmental pseudotime of adult human male germ cells from each iNOA class. Arrows indicate the developmental order of germ cells. Different colors represent different cell types. Peak diagram showing the pseudotime at which each cell type appeared.

Fig. S3

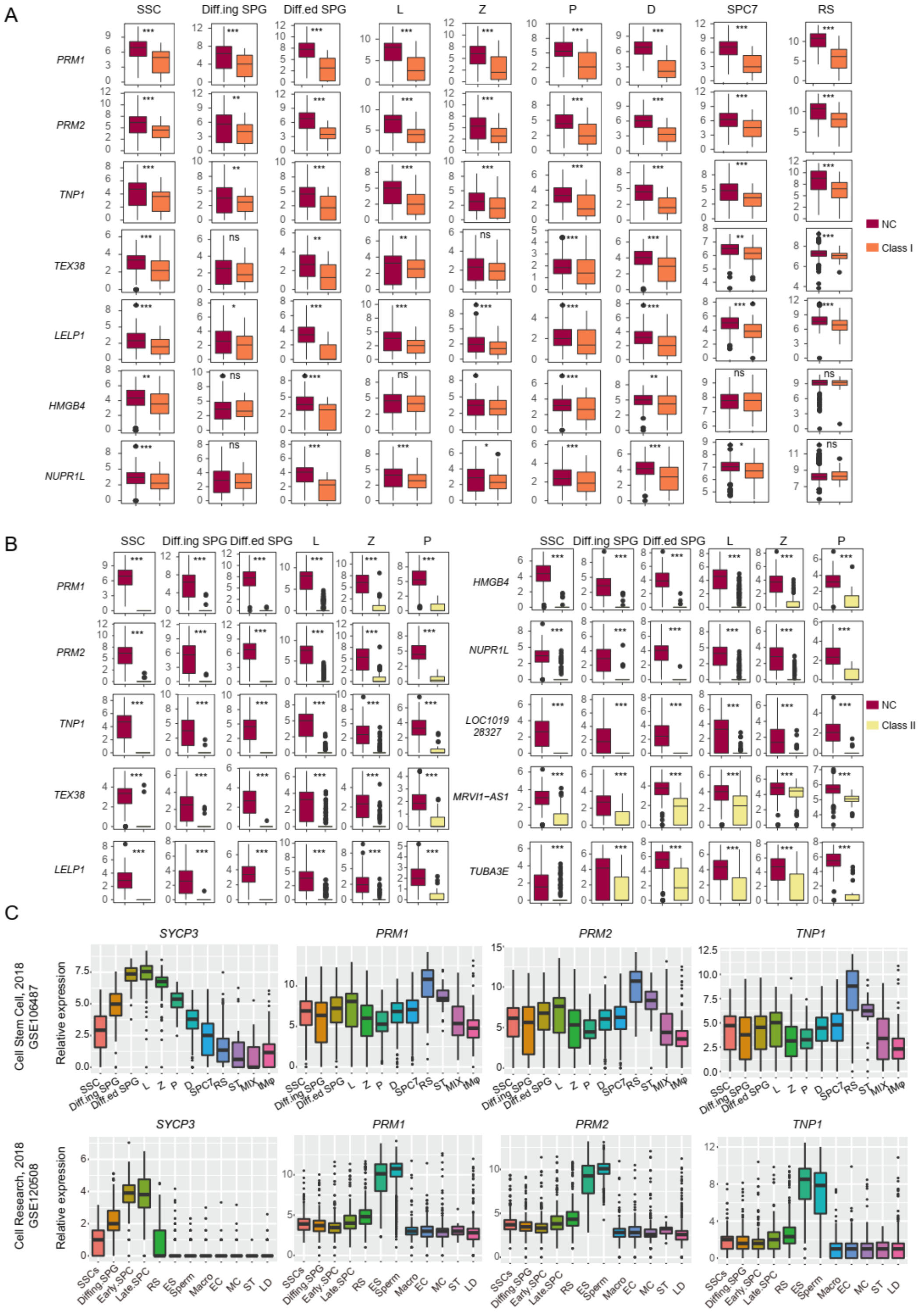

Figure S3. Expression levels of genes significantly altered in iNOA classes I and II. (**A**) Expression levels of the genes significantly downregulated in iNOA Class I compared to the NC group. (**B**) Expression levels of the genes significantly downregulated in iNOA Class II compared to the NC group. The two-tailed

Mann-Whitney-Wilcoxon test was used to assess significance. (**C**) Comparison of expression levels of classical genes in different literature.

Fig. S4

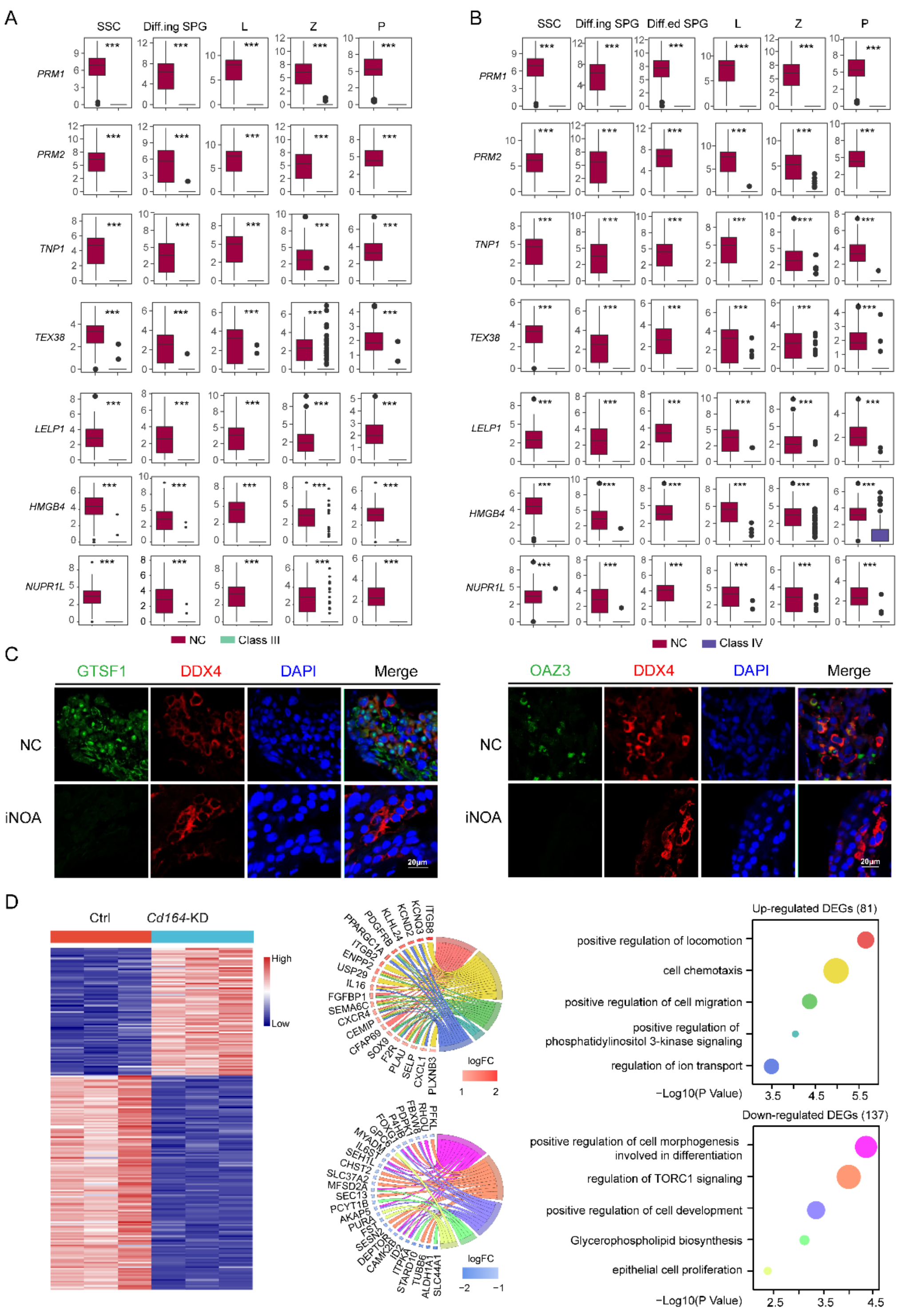

Figure S4. mRNA and protein expression levels of genes significantly altered in

iNOA classes. (**A**) Expression levels of the genes significantly downregulated in iNOA Class III compared to the NC group. (**B**) Expression levels of the genes significantly downregulated in iNOA Class IV compared to the NC group. The two-tailed Mann-Whitney-Wilcoxon test was used to assess significance. (**C**) Immunofluorescence of DDX4 (red) and costaining of target proteins (green) in NC and iNOA. The scale bar represents 20 μm. (**D**) DEGs and associated GO terms in GC1 cells infected with two siRNA.

Fig. S5

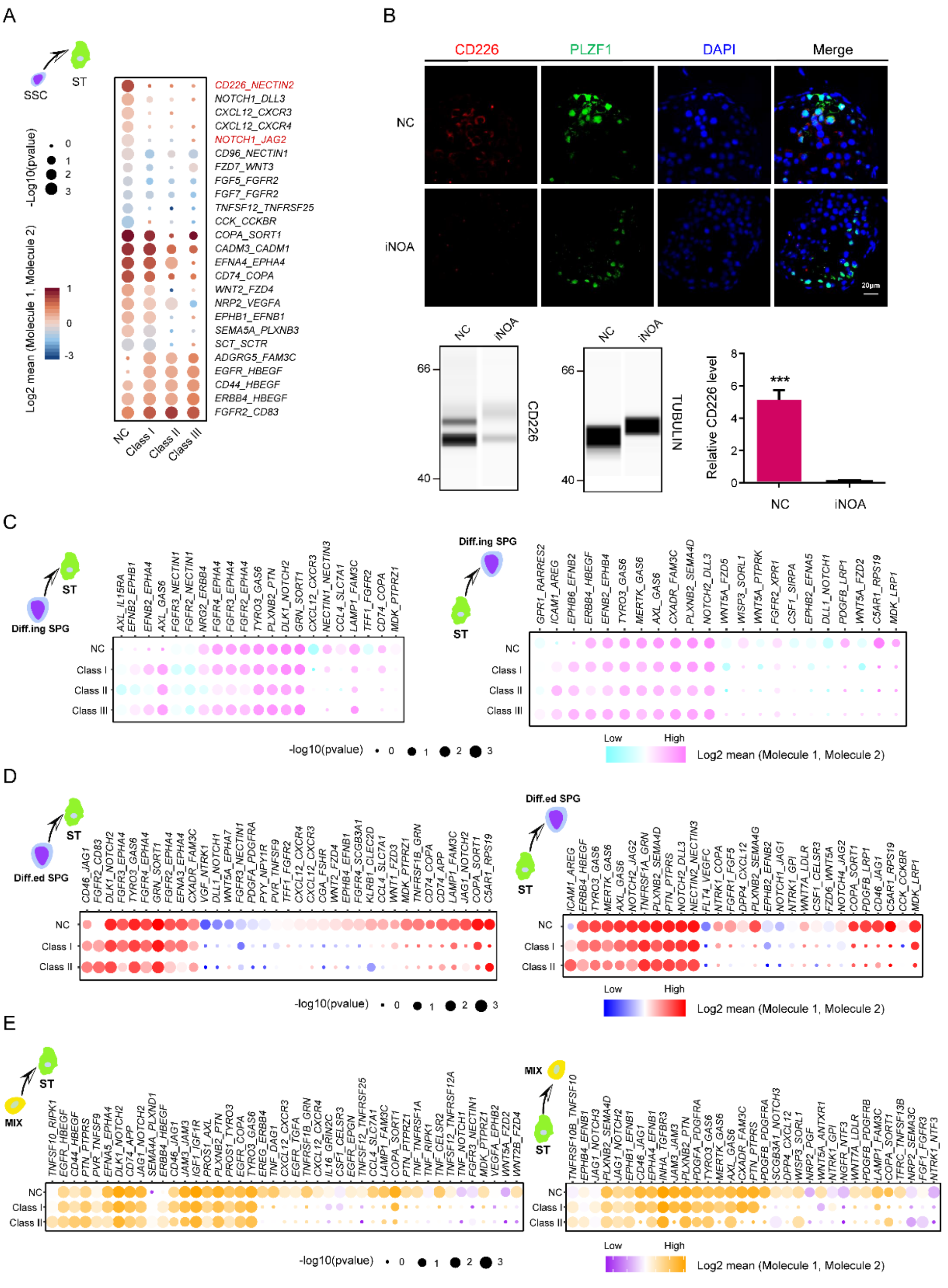

Figure S5. Interactions in spermatogonial cells and somatic cells. (**A**) Interaction of spermatogonial stem cells with ST. Ligands (front) were expressed on spermatogonial stem cells (SSC), and receptors (back) were expressed on ST cells. Dot size represents significance, defined as -$\log_{10}$ (p-value). Color bars from blue to red represent the

normalized expression values of both ligands and receptors. (**B**) Immunofluorescence of CCD26 and the SSC marker PLZF1 in NC and iNOA. The scale bar represents 20 μm. Immunoblotting of CCD26 in testicular tissue of NC and iNOA. Quantification of protein levels is shown. Three samples from each group were analyzed. The Student's t test was used to assess significance. (**C**) Interactions in differentiating spermatogonial cells and Sertoli cells (ST). Dot size represents significance, defined as $-\log_{10}$ (*p*-value). Colored bars represent the normalized expression values of both ligands and receptors. (**D**) Interactions in differentiated spermatogonial cells and ST. Dot size represents significance, defined as $-\log_{10}$ (*p*-value). Color bars represent the normalized expression values of both ligands and receptors. (**E**) Interactions in ST and other somatic cells, mainly Leydig cells. Dot size represents significance, defined as $-\log_{10}$ (*p*-value). Color bars represent the normalized expression values of both ligands and receptors.

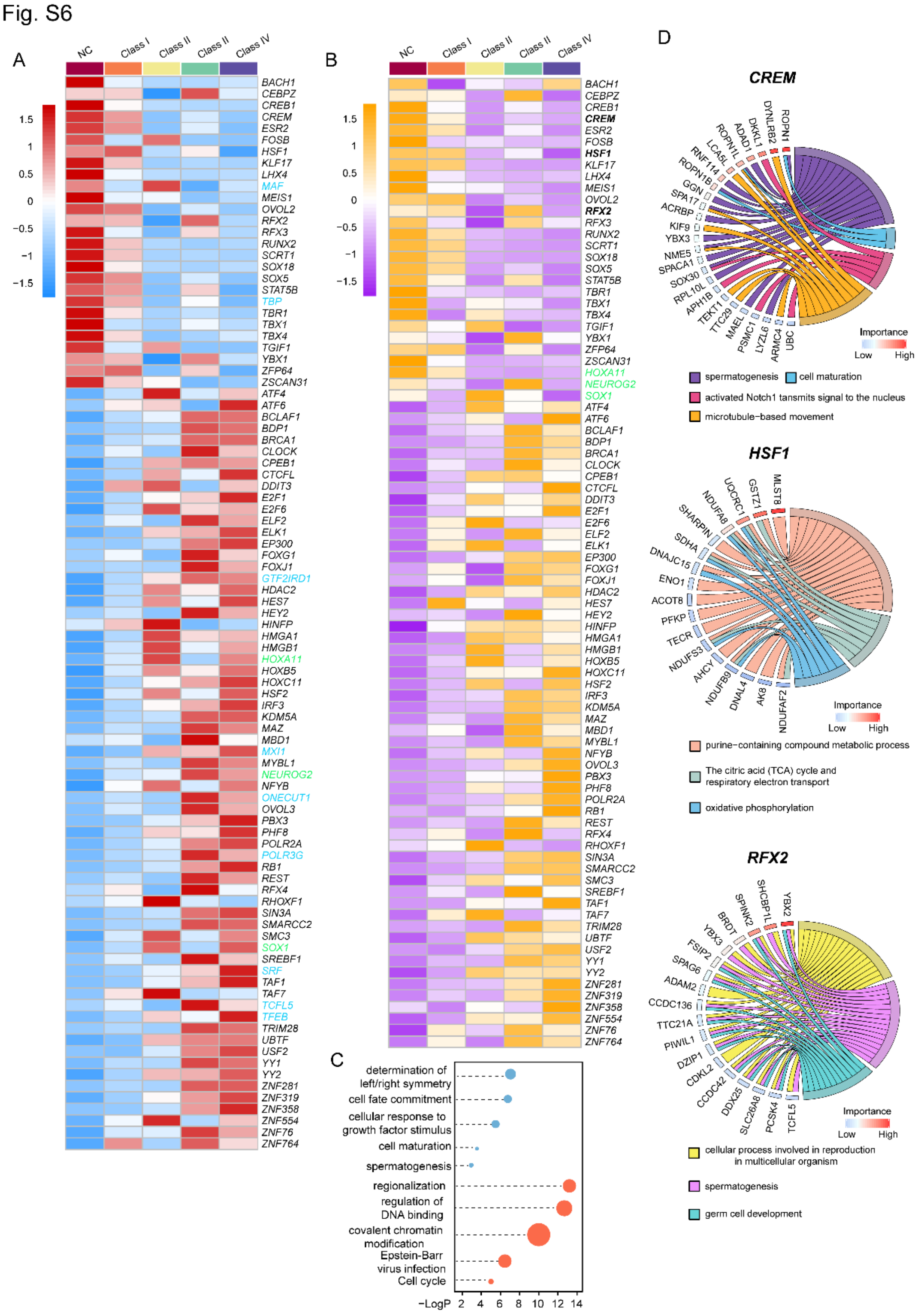

Figure S6. Transcriptional regulatory networks in all germ cells from iNOA samples. (**A**) Transcription factors (TFs) with significantly altered transcriptional activity in iNOA germ cells compared to germ cells from the NC group. TFs whose expression did not significantly differ between iNOA and NC are shown in blue. TFs

with inconsistent changes in transcriptional activity and expression are shown in green. RS cells were excluded in Class II-IV. (**B**) Gene expression patterns of TFs with altered transcriptional activity. TFs with inconsistent changes in transcriptional activity and expression are shown in green. RS cells were excluded in Class II-IV. (**C**) Gene Ontology terms of TFs with altered transcriptional activity. TFs with reduced transcriptional activity and expression are shown in blue. TFs with increased transcriptional activity and expression are shown in red. (**D**) Target genes regulated by *CREM*, *HSF1*, and *RFX2* and the associated signaling pathways. Blue to red indicates low to high significance of the target genes.

Fig. S7

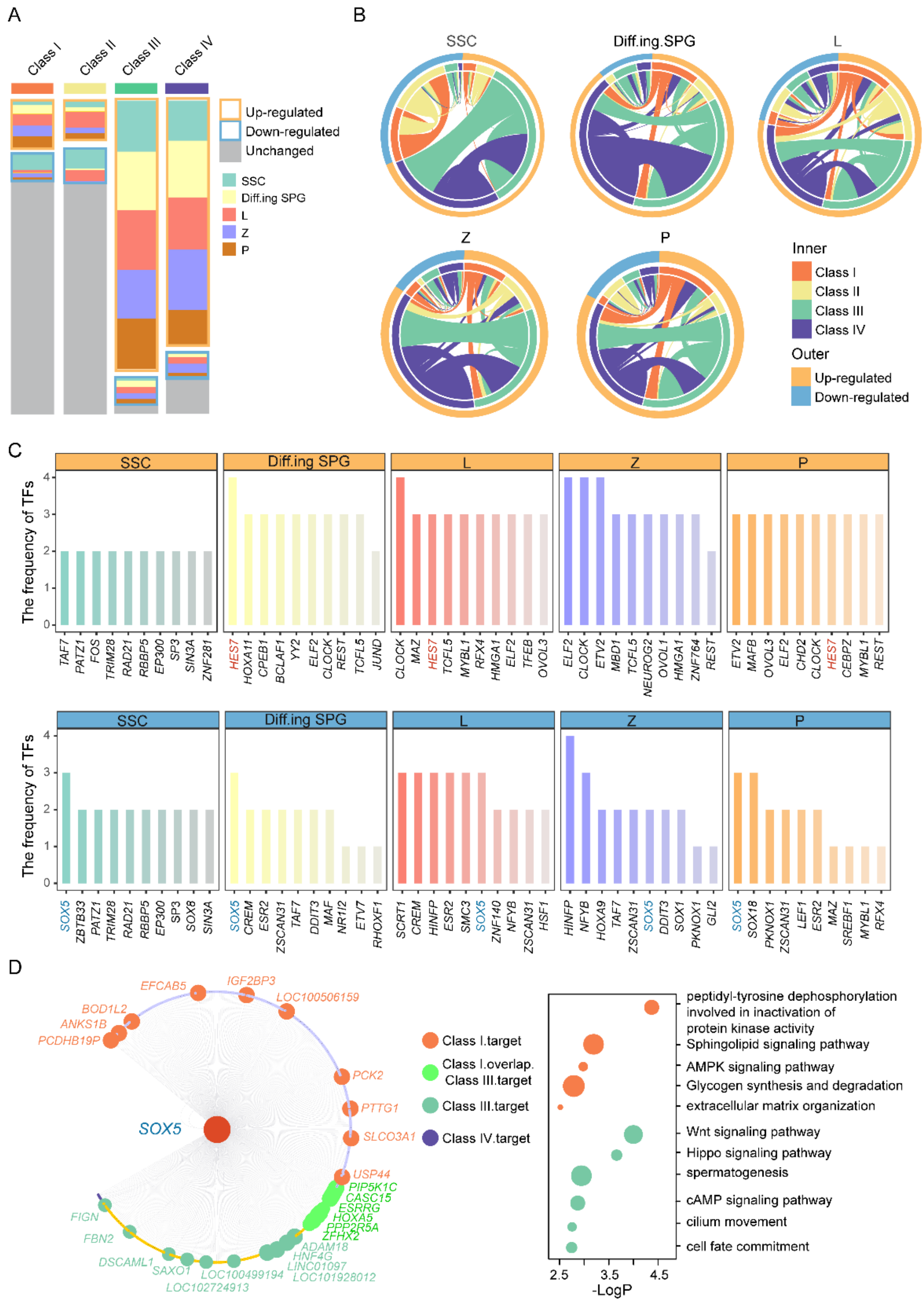

Figure S7. Cell type-specific transcriptional regulatory networks in iNOA. (**A**) Bar plot showing the numbers of cell type-specific upregulated, downregulated, and unchanged TFs in the four classes iNOA compared to the NC group. (**B**) Circos plots showing cell type-specific up and downregulated TFs shared among the four classes

of iNOA. (**C**) Frequencies of TFs in each cell type. The top row shows the cell type-specific upregulated TFs, and the bottom row shows the cell type-specific downregulated TFs. The ordinate (frequency of TFs) represents the number of occurrences in the four classes of iNOA. For example, “3” represents the number of up/downregulated genes in a particular cell type in the three iNOAs. (**D**) Target genes (left panel) regulated by *SOX5* and associated signaling pathways (right panel).

Fig. S8

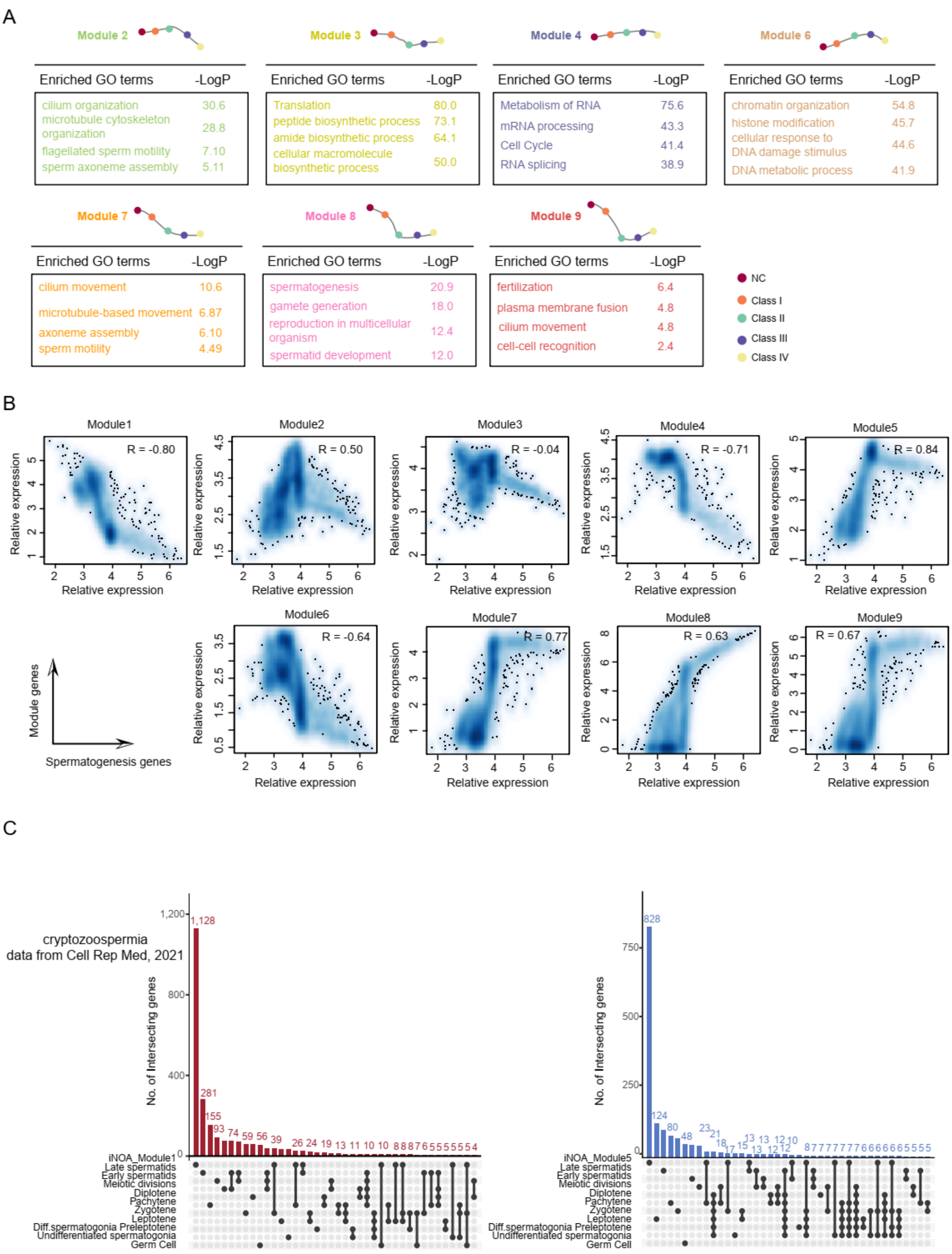

Figure S8. Expression patterns of iNOA trend genes. (**A**) Module genes expression patterns and GO terms. (**B**) Spearman correlation analysis between the module genes and spermatogenic capacity. (**C**) Comparison of iNOA trend genes and DEGs in cryptozoospermia. On the left is the module 1 gene compared to DEGs that are

upregulated in cryptozoospermia patients; on the right is a comparison of the module 5 gene with DEGs downregulated in cryptozoospermia patients. The X axis represents the intersection of module genes and DEGs in testicular disease, compared to NC. The Y axis is the number of intersecting genes.

Supplementary table legends

Table S1. Summary of single-cell RNA-Seq data in 17 idiopathic NOA patients.

Table S2. DEGs of 4 classes in 17 idiopathic NOA patients.

Table S3. GO terms of DEGs in 17 idiopathic NOA patients.

Table S4. Ligand-receptor pairs predicted by CellPhoneDB of all cell types in NC and 4 classes iNOA.

Table S5. Transcription factors predicted by SCENIC in NC and 4 classes iNOA.

Table S6. List of genes predicting testicular spermatogenic capacity.
